# MUTUALITY AMONG EAST ASIAN DIASPORA AT A NATURALLY OCCURRING RETIREMENT COMMUNITY IN METRO VANCOUVER

**DOI:** 10.1093/geroni/igae098.1327

**Published:** 2024-12-31

**Authors:** Daniel Gan, Claire Wang, Eireann O’Dea, Lisa Cohen Quay, Daisy Au

**Affiliations:** University of Toronto, Toronto, Ontario, Canada; Johns Hopkins University, Baltimore, Maryland, United States; Simon Fraser University, Vancouver, British Columbia, Canada; Jewish Community Centre of Greater Vancouver, Vancouver, British Columbia, Canada; MOSAIC, Vancouver, British Columbia, Canada

## Abstract

How did East Asians who were retiring abroad cope with the pandemic? This paper traces the development of a Naturally-Occurring Retirement Community (NORC) in Vancouver through the efforts of various community organizations. As borders closed during the pandemic, a group of community-dwelling East Asian older adults found themselves remaining in Metro Vancouver through the winter. They formed a group chat and began regular video chats. They shared virtual events, supermarket deals, and neighbourhood walks. A younger member stepped up to ask after members of the informal group whom were absent or unwell. Topics of discussions ranged from the wellbeing of members and their spouses, securing medical appointments, translating forms to be filled, friends back home, leisure activities, and the lives of their children or grandchildren. The group was formally served by facilitators of a seniors’ club at a non-profit. At the same time, they existed as an informal network marked by mutual care. We discuss the health-promoting nature of mutuality in this NORC as compared to other neighbourhoods to theorize the conditions needed for the emergence of Gemeinschaft NORC in urban areas. This paper sketches a theory of mutuality as the key which transforms dyadic peer support into community cohesion. We explore the implications of mutuality on formal programs in order to harness their potential to have population-level spillover effects for the wellbeing of community members just beyond their direct reach. We further speculate that mutuality is that which was lost in modernity, and is the reason culture is medicine.

